# RETRACTION: Hsp90‐binding immunophilin FKBP51 forms complexes with hTERT enhancing telomerase activity

**DOI:** 10.1002/1878-0261.13676

**Published:** 2024-06-05

## Abstract

RETRACTION: M. Lagadari, N. R. Zgajnar, L. I. Gallo and M. D. Galigniana, ‘Hsp90‐binding immunophilin FKBP51 forms complexes with hTERT enhancing telomerase activity’, *Molecular Oncology* 10, no. 7 (2016): 1086–1098, https://doi.org/10.1016/j.molonc.2016.05.002.

The above article, published online on 17 May 2016 in Wiley Online Library (wileyonlinelibrary.com), has been retracted by agreement between the journal Editor in Chief, Kevin Ryan and John Wiley and Sons Ltd. The retraction has been agreed due to several instances of image manipulation in figures 1C, 5B, 4A and 5A. The available raw data were not compelling. Based on the number of inappropriate duplications, the editors consider the conclusions substantially compromised and are therefore retracting the paper. The author, Mario D. Galigniana, disagrees with the retraction; acknowledgement of the retraction could not be obtained from the remaining co‐authors.

RETRACTION: M. Lagadari, N. R. Zgajnar, L. I. Gallo and M. D. Galigniana, ‘Hsp90‐binding immunophilin FKBP51 forms complexes with hTERT enhancing telomerase activity’, *Molecular Oncology* 10, no. 7 (2016): 1086–1098, https://doi.org/10.1016/j.molonc.2016.05.002.

The above article, published online on 17 May 2016 in Wiley Online Library (wileyonlinelibrary.com), has been retracted by agreement between the journal Editor in Chief, Kevin Ryan and John Wiley and Sons Ltd. The retraction has been agreed due to several instances of image manipulation in figures 1C, 5B, 4A and 5A. The available raw data were not compelling. Based on the number of inappropriate duplications, the editors consider the conclusions substantially compromised and are therefore retracting the paper. The author, Mario D. Galigniana, disagrees with the retraction; acknowledgement of the retraction could not be obtained from the remaining co‐authors.

